# Extending the Protection Ability and Life Cycle of Medical Masks through the Washing Process

**DOI:** 10.3390/ma16031247

**Published:** 2023-02-01

**Authors:** Julija Volmajer Valh, Tanja Pušić, Mirjana Čurlin, Ana Knežević

**Affiliations:** 1 Faculty of Mechanical Engineering, University of Maribor, Smetanova 17, 2000 Maribor, Slovenia; 2 Faculty of Textile Technology, University of Zagreb, Prilaz Baruna Filipovića 28a, 10000 Zagreb, Croatia; 3 Faculty of Food Technology and Biotechnology, University of Zagreb, Pierottijeva 6, 10000 Zagreb, Croatia

**Keywords:** medical masks, washing, detergent, didecyldimethylammonium chloride, air permeability, antimicrobial activity, residuals

## Abstract

The reuse of decontaminated disposable medical face masks can contribute to reducing the environmental burden of discarded masks. This research is focused on the effect of household and laboratory washing at 50 °C on the quality and functionality of the nonwoven structure of polypropylene medical masks by varying the washing procedure, bath composition, disinfectant agent, and number of washing cycles as a basis for reusability. The barrier properties of the medical mask were analyzed before and after the first and fifth washing cycle indirectly by measuring the contact angle of the liquid droplets with the front and back surface of the mask, further by measuring air permeability and determining antimicrobial resistance. Additional analysis included FTIR, pH of the material surface and aqueous extract, as well as the determination of residual substances—surfactants—in the aqueous extract of washed versus unwashed medical masks, while their aesthetic aspect was examined by measuring their spectral characteristics. The results showed that household washing had a stronger impact on the change of some functional properties, primarily air permeability, than laboratory washing. The addition of the disinfectant agent, didecyldimethylammonium chloride, contributes to the protective ability and supports the idea that washing of medical masks under controlled conditions can preserve barrier properties and enable reusability.

## 1. Introduction

The challenges of the COVID-19 epidemic/pandemic and the efforts against the spread of the virus require both short- and long-term recommendations and measures. One of the recommendations and types of personal protection is wearing a mask. In practice, three types of masks with different characteristics and degrees of protection have become widely used, namely face masks, medical masks, and filtering masks. Their characteristics and protective effects are usually highlighted in the attached product specifications. The face mask can prevent the spread of infectious diseases in public areas by preventing both the inhalation of infectious droplets and their subsequent exhalation and transmission [1]. 

Home textiles have been shown to play an important role in spreading infections caused by pathogenic microorganisms: viruses, bacteria, and fungi [2,3,4].

The results of the research show that cleaning and washing textiles is one of the key factors in ensuring hygiene in the home [5,6,7]. There are other types of disinfection, e.g., ultraviolet (UV) light with destructive activity against pathogenic bacteria, including Clostridioides difficile spores [8], hydrogen peroxide, UV-rays, moist heat, dry heat, and ozone [7].

The main aim of household laundering is to remove visible and invisible dirt and unpleasant odors to achieve freshness, a satisfactory aesthetic appearance, and a high level of cleanliness. This can be achieved by optimizing the factors of the Sinner’s circle, compensating for an increase in the proportion of one of the factors by reducing the others [9,10]. The key factor in decontamination of microorganisms on surfaces is increased temperature. Considering the environmental guidelines to lower the washing temperature and the increased proportion of synthetic textiles, the chemical effect based on the composition of the detergent is of utmost importance. This makes bleaching agents and their activators important factors for disinfection during washing [11,12]. In applying disinfectants, the principle of efficiency is the most important requirement, while at the same time the principle of sustainability and environmental performance must be observed.

Numerous challenges and the pandemic period of SARS-CoV-2 affecting people’s respiratory system [13] have raised certain questions and doubts about the extent to which consumer laundry detergents can reasonably ensure the level of disinfection during washing. There are also questions about the extent to which washing and rinsing aids can improve disinfection efficacy [14,15].

During the global COVID-19 pandemic, cationic surfactants were extensively used as antiseptics and disinfectants [16]. Recent studies have shown that the growth in the production and use of surfactants was +196%, for biocides it was +152%, and for cationic quaternary ammonium surfactants (used as surfactants and biocides) it was +331% [17].

Research has confirmed that disposable face masks belong not only to personal protective equipment (PPE) that greatly contributes to the protection of people [18,19,20] but also to the generation of textile waste [21,22]. The problem of the accumulation of textile waste gave rise to the idea of reusing medical masks after decontaminating them in the process of washing them with detergents at 60 °C and treating them with disinfectants, such as hydrogen peroxide, UV rays, moist heat, dry heat, ozone, ethanol, and sodium hypochlorite [7,19,20,23,24]. Decontamination procedures, along with effective pathogen reduction, should preserve the protective features of medical masks (filtration efficiency and breathability) without the harmful chemical effect on the user [18,19].

In this research, the influence of household and laboratory washing procedures on the properties of disposable medical masks made of three-layer nonwoven polypropylene was investigated. The novelty of this investigation can be seen in several aspects. The first is the reduction in the washing temperature from 60 °C to 50 °C. Washing the masks with commercial color detergent at home and in the laboratory with standard detergents to verify reusability and extend the life cycle distinguishes this investigation from others. In addition, the influence of the factors of the washing process at home and in the laboratory on the degree of protection against respiratory droplets and the human ecological properties of disposable protective masks was investigated. Washing in the laboratory was performed with a standard detergent, and the rinsing process was varied. After washing with detergent, part of the medical mask samples was rinsed with water in four cycles, while the remaining samples were rinsed with water in three cycles, and disinfection with didecyldimethylammonium chloride (DDAC) was performed in the fourth cycle.

The barrier properties of the medical mask—the effectiveness of “protection against respiratory problems” before and after the first and fifth washing cycle in the specified conditions were analyzed indirectly by measuring the contact angle of the liquid droplets with the front and back surface of the mask, measuring air permeability, and determining antimicrobial resistance.

The reusability and washability of medical masks were analyzed by FTIR, by measuring the pH of the material surface and the aqueous extract. The new concept for characterization of masks includes the analysis of surfactants in an aqueous extract, which may have irritating potential. The aspect of the aesthetic component of the masks was studied by measuring their spectral properties.

## 2. Materials and Methods

Disposable medical face masks, model KZ020, manufactured by Chuzhou Qiao Dong Industrial Co., Ltd, (Quzhou, China) were purchased through the distribution channel of Drogerie Markt (dm), Coburg, Germany. The nonwoven structure of the masks is made of polypropylene fibers (PP), with the proportion of PP microfibers of 30%, produced by meltblown technology and integrated between two spunbond layers [25]. The material is declared as hypoallergenic, and the mask is classified as type IIR, whose filtration efficiency is 98%, and the properties meet the requirements of HRN EN 14683:2020. The front surface of the medical mask is blue, and the back is white, with ear loops and a reinforcement in the area of the nose (nose wire).

The masks were washed in a Hanseatic household washing machine, model HWT 8614A, at 50 °C, with a liquid color detergent (100 g) containing anionic surfactants (5–15%), nonionic surfactants (≤5%), phosphonates, polycarboxylates, zeolites, enzymes, fragrance hexyl cinnamal, and water. After the washing cycle, 4 rinsing cycles with inter-centrifugations according to the washing machine program were performed. The described procedure was repeated 5 times.

The washing in the laboratory was performed in a laboratory device PolyColor, Werner Mathis AG, Oberhasli, Switzerland, at 50 °C with a standard ECE A detergent containing linear sodium alkylbenzene sulfonate (9.7%), sodium soap (5.2%), defoamer (4.5%), sodium aluminosilicate (32.5%), sodium carbonate (11.8%), sodium salt copolymer of acrylic and maleic acid (5.2%), sodium silicate (3.4%), carboxymethyl cellulose (1.3%), diethylenetriamine penta (methylene phosphonic acid 5.2%), sodium sulfate (9.8%), and water (12.2%).

The detergent was dosed in a concentration of 1.25 g/L with a bath ratio of 1:50.

After the washing process in the laboratory device, the medical mask samples from the container with plastic tweezers were transferred to a laboratory beaker, where they were rinsed in two ways: (i) 4 individual rinsing cycles in water; (ii) 3 individual rinsing cycles in water, and subsequent disinfection in water with 1 mL/L of disinfectant for 30 min as recommended by the supplier. The main ingredient of this disinfection agent is the cationic active substance, didecyldimethylammonium chloride (DDAC, according to IUPAC, N-decyl-NN-dimethyldecane-1-ammonium chloride). It is specified that 100 g of this agent contains 2.49 g of DDAC.

After the individual washing and rinsing, the samples were air dried in a protected area and placed in plastic zip bags to avoid possible contamination. The sample labels before and after the 1st (1×) and 5th (5×) washing according to the described procedures are shown in Table 1.

### 2.1. Methods

The medical masks listed in Table 1 were analyzed by the methods for the purpose of monitoring their properties. Fourier transform infrared spectroscopy was used to characterize the composition of the medical masks. Spectrum 100S FT-IR UATR + TG /IR Interface TL8000 (RedShift), Perkin Elmer, Waltham, MA, USA was used for the analysis.

The spectral characteristics of the front of the medical masks were measured at four different locations using a Spectraflash SF300 computer-controlled dual-channel remission spectrophotometer, Datacolor AG, Rotkreuz, Switzerland, with an aperture of 20 mm, standard light D65, and d/8° geometry. Results are presented as differences in lightness (dL*), hue (dH*), and chroma (dC*) of the washed samples compared to the pristine samples.

The total color difference (*dE*) of the washed samples compared to the original medical mask sample is calculated as follows in Equation (1):

(1) $$dE=\sqrt{{\left(dL^{*}\right)}^{2}+{\left(dC^{*}\right)}^{2}+{\left(dH^{*}\right)}^{2}}$$

dL*—difference in lightness (dL* = L*washed sample − L*pristine)

dC*—difference in chroma (dC* = C*washed sample − C*pristine)

dH*—difference in hue

Instrumental evaluation of color change to determine the gray scale rating was additionally performed according to the rating system ISO A05 and AATCC. The method also specifies an instrumental method for evaluating the color change in a washed mask compared to an untreated mask as a reference. Calculations were performed to convert the instrumental measurements into a gray scale rating, with a score of 5 indicating excellent color fastness and a score of 1 indicating poor color fastness [26].

The whiteness of the back surface of medical masks before and after washing was determined by spectral measurement (Spectraflash SF300, Datacolor AG, Rotkreuz, Switzerland), with an aperture size of 20 mm, under standard illumination D65) according to ISO 105-J02:1997 Textiles—Colour fastness testing—Part J02: Instrumental assessment of relative whiteness [27]. The measurement results were expressed as the average of four individual measurements.

The analysis of the presence of aerobic bacteria on medical mask samples was performed according to the standard procedure for water analysis [28].

This standard method was adapted to a medical mask sample inoculated onto microbiological agar in a Petri dish. Samples were incubated at a temperature of 37 °C for 48 h or at a temperature of 22 °C for 72 h. After incubation, all colonies present in each inoculated dish were determined visually. Since this method was modified and adapted to mask samples, the presence of colonies on the plates was taken as a visual result only and is presented by images for the unwashed sample the home-washed sample and the sample washed under laboratory conditions and rinsed with a disinfectant.

After determining the contact angles of the liquid droplets with the surface of the medical masks using a goniometer (DataPhysics, Filderstadt, Germany), the hydrophilicity/hydrophobicity of the samples was evaluated before and after the washing cycle. The static contact angle (SCA) was measured with a drop of liquid lying on the surface. At least 3 measurements were taken for each sample, and even more individual measurements were taken for some samples. A goniometer with OCA 20 software was used to determine the SCA at room temperature with ultrapure water. A drop with a volume of 3 µL was carefully placed on the surface of the sample.

The barrier properties of medical masks were established indirectly by determining the air permeability according to EN ISO 9273 (Textiles—Test methods for nonwovens—Part 15: Determination of air permeability) to evaluate the influence of washing conditions. To measure air permeability, a device with four working heads produced by the Karl Schroeder company was used (power supply U = 220 V; frequency f = 50 Hz; current I = 5 A; power P = 1100 W; with a three-phase measurement range: 5...90 L/m²xs, 50...550 L/m²xs, 500...5600 L/m²s). The air permeability of the medical masks before and after the process was measured at three different points (at the center of the mask and two measurements at a radius of 1 cm around the center of the mask).

The nonwoven structure of the front and back surface of the medical mask was characterized by measuring the pH of the sample at four different locations using the contact electrode of the Multimeter SevenCompact™ Duo S213 (Mettler-Toledo GmbH, Greifensee, Switzerland).

The pH of the aqueous extract of the nonwoven structure was determined in accordance with EN ISO 3071:2020 (Textiles—Determination of pH of aqueous extract) such that the elastics (ear loops) and flexible nose piece were removed from the samples. For this analysis, 6 samples were excluded, half of which were cut into strips of 5 mm (C), while the remaining 3 were left intact (N). The procedure was performed by placing 2 g of the sample in an Erlenmeyer flask and adding 100 mL of KCl solution. The flasks were exposed to mechanical shaking for 2 h. After mixing, the pH of the aqueous extract was measured using the previously described multimeter.

Considering the composition of detergents in washing and of DDAC in rinsing, the content of residual surfactants in the aqueous extract of the medical masks was analyzed: anionic and nonionic ones from detergents and cationic ones from DDAC. Potentiometric titrations with the Metrohm Autotitrator 736 GP Titrino and electrodes, all from Metrohm, Herisau, Switzerland, were used to determine the residual surfactants. For the determination of anionic and cationic surfactants, the High Sense Surfactant Electrode (6.0504.150) was used as an ion-selective surfactant electrode in combination with the reference, the Ag/AgCl electrode (6.0733.100). The NIO surfactant electrode (6.0507.010) in combination with the same reference electrode was used for the determination of the nonionic surfactant. The determination of a surfactant residue in the aqueous extract by potentiometric titrations was preceded by the preparation of calibration curves. Selected residues: (i) solutions of the anionic surfactant sodium dodecyl sulphate (NLS) were titrated with Hyamine 1622 at pH 3; (ii) solutions of the cationic surfactant (Hyamine 1622) were titrated with NLS at pH 10; (iii) solutions of the nonionic surfactant (Triton X-100) were titrated with sodium tetraphenyl borate and the addition of BaCl2 [29,30].

## 3. Results and Discussion

### 3.1. Medical Masks Characterization

#### 3.1.1. Spectral Characteristics

Medical masks have different colors; the front surface is blue and the back is white. Accordingly, the change in spectral characteristics of the front surface was monitored by the differences in lightness (dL*), chromaticity (dC*), hue (dH*), total color difference (*dE*), and color fastness grades of laboratory and home-washed versus unwashed masks according to ISO and AATCC, Table 2.

The changes in spectral properties of medical masks after the first and fifth washing depend on the washing conditions. The household washing was carried out with a liquid color detergent and the laboratory washing with a standard detergent. These detergents have different compositions, which consequently affects the pH of their solutions. The results of the total difference in color (*dE*) indicate that the effect of the first household wash (H) is slightly weaker compared to the laboratory washing for both variants (L and L_DS_). However, the cumulative effect of the household washing over five cycles is more noticeable (rating 3) than that of the laboratory washing (rating 4), which can be attributed to mechanical agitation, including interval centrifugation in the washing machine [31]. The laboratory wash with the addition of DDAC in the final rinse cycle (L_DS_) has a mild protective effect on the hue, as evidenced by the lower dE values and color fastness ratings compared to the laboratory wash alone (L).

The back of the medical mask is a white nonwoven structure. The influence of washing was analyzed in terms of whiteness (W_CIE_ and Y) and hue (TV, TD), with the average values shown in Table 3.

The baseline whiteness of the back side of an unwashed medical mask is 98.5, indicating that no optically active components are present. Household washing was performed with a liquid color detergent containing no optical bleaching agents, to which only slightly different values for the whiteness of the medical mask (H) were indicated.

Laboratory washing was performed with a standard powder detergent that also did not contain optical brightening agents. There is a decrease in whiteness by more than 10 units for the samples washed 1× and 5× compared to the unwashed sample. These results may indicate the presence of residual substances from the laboratory washing process for both variants.

#### 3.1.2. Antimicrobial Properties

To achieve the antimicrobial activity of the synthetic fibers, of which the tested medical masks are made, an antimicrobial agent can be incorporated into the polymer before spinning or mixed into the fibers during manufacture. This ensures maximum durability, as the agent is physically embedded in the fiber structure and is released at a certain rate during use. Since no antimicrobial agents embedded in the analyzed medical masks were either known or detected, the antimicrobial efficacy using the usual and standardized method for textile materials was not determined.

The disinfectant agent contained a cationic active component, didecyldimethylammonium chloride (DDAC). This agent, from the supplier dm GmbH, was dosed into the final rinse water, where it was left to sit for at least 30 min. According to the manufacturer the described procedure removes 99.9% of bacteria, fungi, and viruses. This agent has been dermatologically tested, does not affect the feel of textiles, and has no irritating effect on the skin. However, the supplier points out that people with sensitive skin must strictly follow the dosage instructions.

**Figure 1:**
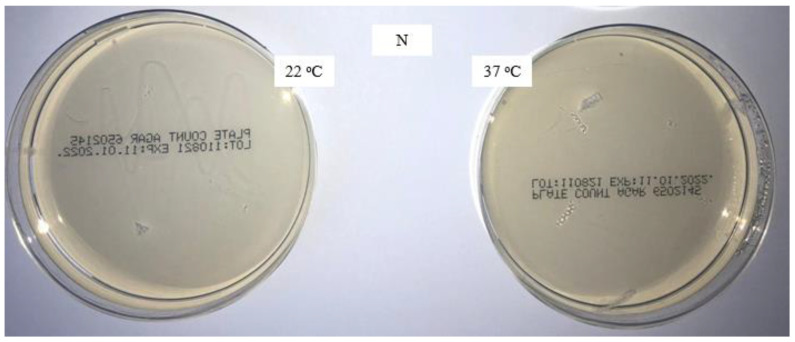
Colonies of aerobic microorganisms on a pristine medical mask (N).

**Figure 2:**
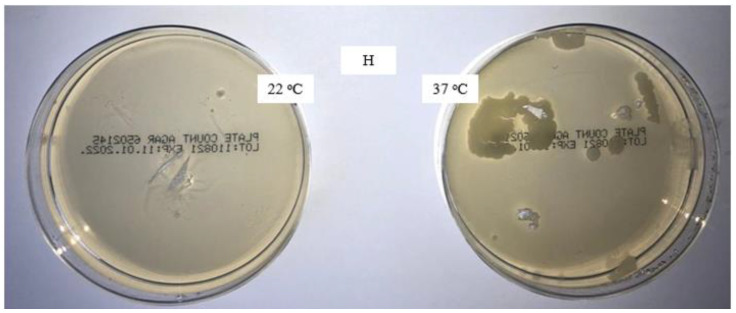
Colonies of aerobic microorganisms on a medical mask after household washing (H 5×).

**Figure 3:**
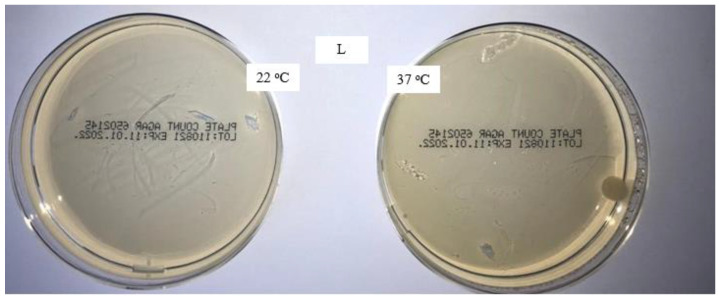
Colonies of aerobic microorganisms on a medical mask after laboratory washing (L 5×).

**Figure 4:**
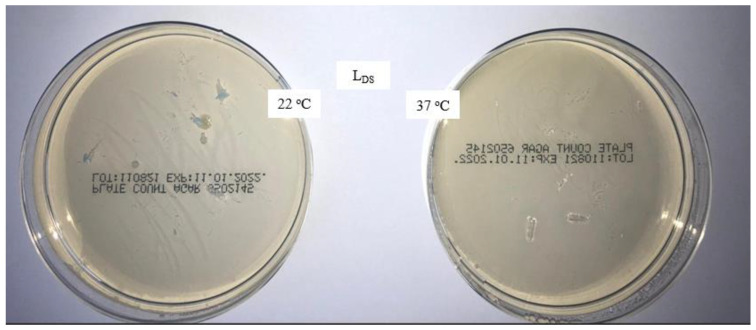
Colonies of aerobic microorganisms on a medical mask after laboratory washing with the addition of DDAC (L_DS_ 5×).

The aim of this study was to verify the effect of the disinfectant DDAC and to compare the microbiological characteristics of unwashed masks, masks washed in the household with detergent, and masks washed in the laboratory with detergent only and detergent with the addition of DDAC. The presence of colonies of aerobic microorganisms on all samples was tested with the method for the microbiological analysis of water, adapted to the rigid sample of masks. The analysis results after incubation for 72 h at 22 °C and for 72 and 48 h at 37 °C are shown in Figure 1, Figure 2, Figure 3 and Figure 4.

Figure 1 shows that no aerobic bacteria were present after incubation at 22 °C and 37 °C, indicating a certain purity (sterility) of the purchased masks, which were packaged in a plastic bag.

On a medical mask washed in household conditions, a certain number of colonies of aerobic microorganisms was detected (Figure 2). The reason for the appearance of microorganisms was the fact that the masks were washed in the washing machine together with the other laundry, so that it is assumed that a cross-contamination occurred either from other textiles or from contamination in the washing machine.

Figure 3 shows the presence of microorganisms on medical masks washed in the laboratory with a standard detergent. The amount of microorganisms was smaller compared to medical masks washed at home. These samples point to a contamination of the washing system, or the ineffectiveness of the standard detergent for this activity.

The addition of the disinfectant DDAC in the fourth rinse cycle has a significant effect on preventing the appearance of microorganisms on the mask samples washed in laboratory tests, as can be seen in Figure 4, where no colonies of microorganisms are visible.

The final results regarding the presence of microorganisms on samples of unwashed and washed masks after different procedures point to the disinfecting effect in the washing process with DDAC. These results justify the washability of medical masks at 50 °C through five cycles and the possibility of reuse. These results are comparable to the functional properties of medical masks washed at 60 °C [20] with the addition of a disinfectant [7,32], whose ecological performance is questionable.

Further research will be focused on testing the effectiveness of DDAC in household washing where, based on the results, it is necessary to prevent cross-contamination of the system.

#### 3.1.3. Hydrophobic Properties

The behavior of droplets on the textile surface (shape, spilling, and sorption) depends on the surface, the type of liquid, the interaction of cohesive forces (between liquid molecules), and adhesive forces (between fibers and liquid) [33,34]. Medical masks are waterproof to a certain extent [35]. The influence of washing conditions on the changes in the adhesive properties of the front and back surface of the medical mask was analyzed by determining the static contact angle (SCA). The average values of the SCA on the front and back surfaces are highlighted in Table 4 and Table 5. In determining the SCA on all the samples of the medical masks, there was the problem of wrinkles on the unwashed samples and breaks on the washed samples due to the mechanical agitation in the washing process.

The average SCA of the unwashed medical mask (N) was 131.24°, which demonstrates the expected hydrophobicity of the medical mask, considering that it is made of polypropylene (PP). The values of the individual measurements of the unwashed sample were considerably scattered, which could be expected given the fact that the analyzed structure is nonwoven.

The influence of washing on the SCA depended mainly on the number of cycles performed, again with a large scattering of the individual measurements. In all washing processes the contact angle of the samples was smaller after the first wash cycle than after the fifth one. The reason for this could be the deposition of inorganic substances, as the washing process was carried out in hard water.

The result analysis of the SCA of the liquid droplets with the back of the medical mask proves the previously highlighted differences in the individual measurements. The changes in the SCA of the droplets with the back do not completely comply with the number of cycles on the contact angle of the droplets with the front of the medical mask.

**Figure 5:**
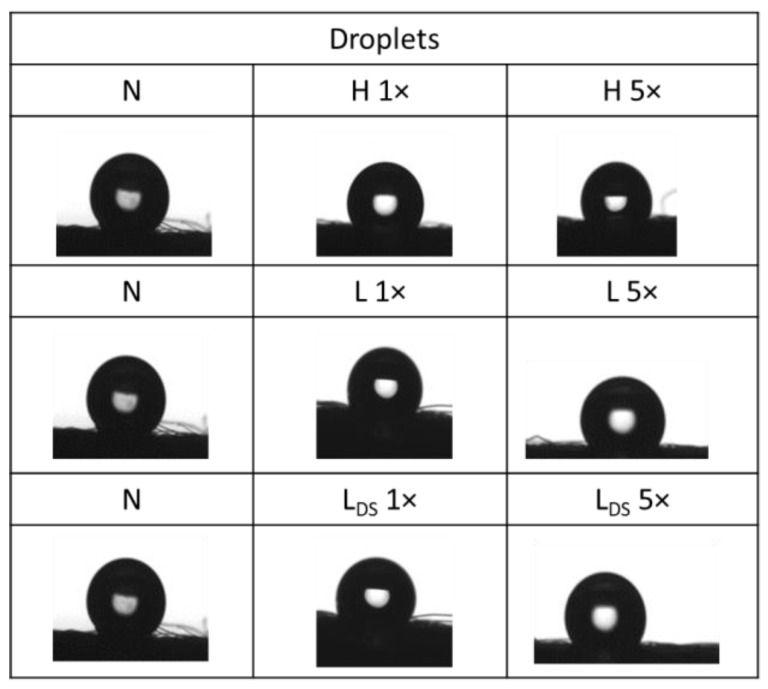
Droplets of the liquid with the front surface of the medical mask before and after washing.

**Figure 6:**
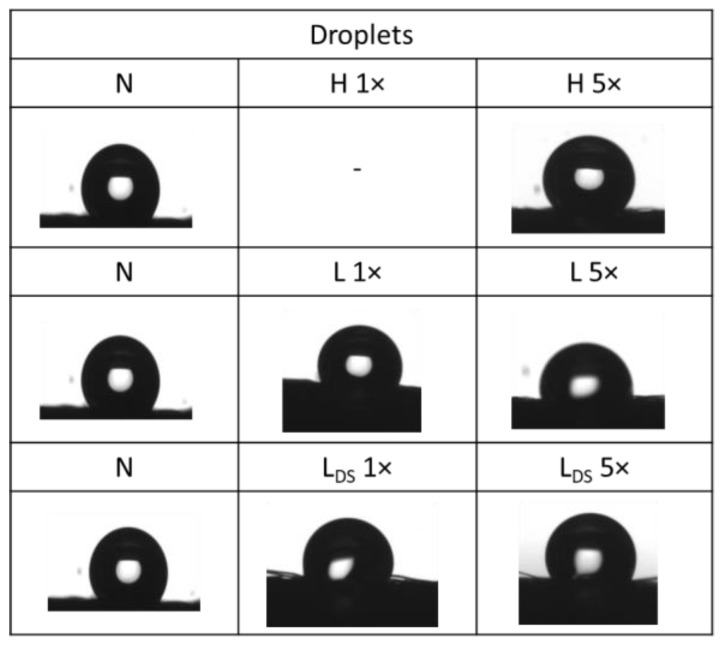
Droplets of the liquid with the surface of the back surface of the medical mask before and after laboratory washing with a standard detergent and rinsing with a disinfectant.

The photographs of the liquid droplets with the surface of the medical mask sample are shown in Figure 5 and Figure 6.

The pictures of droplets confirm the hydrophobicity of the front/back surface of the medical mask before and after washing. There are differences in the appearance of the surface of the examined samples, in the form of protruding fibers. Their appearance from the nonwoven structure is to be expected, while their distribution depends on numerous parameters of washing, drying, and testing.

#### 3.1.4. Air Permeability

Information about air permeability properties of certain textiles makes it possible to evaluate and compare the performance characteristics of specific products, such as raincoats, tents, shirting fabrics, sails, industrial filters, and pillowcases. Testing this property is important when a softener is included in the finish or care [36,37]. The use of different types of softeners causes significant differences in air permeability [38].

Air permeability was tested according to EN ISO 9237 (Textiles—Determination of air permeability of fabrics). According to this source, the air permeability should be at least 96 L/m^2^s at a vacuum pressure of 100 Pa [39].

The air permeability of medical masks measured on samples before and after the washing cycles is shown in Table 5. 

The air permeability of an unwashed medical mask at a pressure of 1013 mbar (≈105 Pa) was 31.0 L/m^2^s. The air permeability of the washed samples in the wash processes (H and L) increased compared to the unwashed sample. The increase in air permeability of the samples washed at home was greater than in the laboratory. The values of the samples washed at home (H 1x) increased six and three times (H 5x) compared to the unwashed sample (N). The differences in air permeability of the medical mask samples after the first and fifth cycle of washing in the laboratory (L and L_DS_) were insignificant; they were about three times higher than for the unwashed sample (N).

#### 3.1.5. pH Analysis

The pH analysis of textiles after various processing and care procedures is useful because it provides information about the condition of the surface. In this work, the pH of the front and back surfaces of unwashed and washed medical mask samples was tested using a contact electrode (Table 6 and Table 7).

The front surface of the unwashed mask had a pH of 5.79. This value was almost the same for the home-washed samples, which was expected given the lower pH of the color liquid detergent solution. Washing under laboratory conditions for five cycles resulted in an increase in the pH of the surface (pH ~ 7.00), owing to the standard detergent, the solution of which is strongly alkaline (pH ~ 10.50).

The changes on the back surface were similar to the changes on the front of the medical mask, which was expected given the washing conditions.

#### 3.1.6. FTIR Analysis

**Figure 7:**
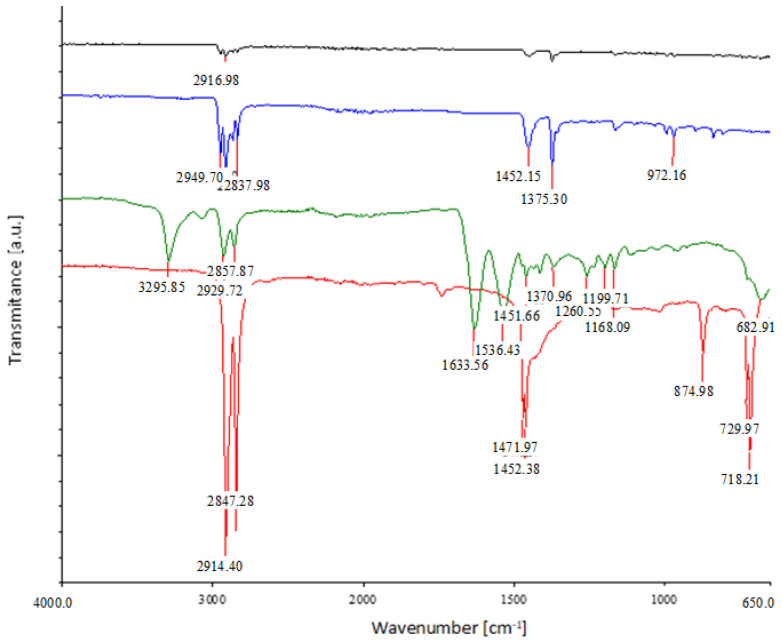
FTIR spectra of the back (black spectrum) and front (blue spectrum) nonwoven layer, the ear loops (green spectrum), and nose pads (red spectrum) of the medical mask.

The pristine (N) medical mask was characterized by FTIR spectroscopy to confirm the composition of the raw material (Figure 7). The spectra of the inner (back) and outer (face) nonwoven layers of the medical mask, the ear loops, and nose pads were recorded. 

The spectra of the inner (back) and outer (face) layers showed the following bands: multiple signals in the wavenumber range from 3000 to 2800 cm^−1^ and two large signals in the range from 1452 to 1375 cm^−1^. The signals in the range from 3000 to 2800 cm^−1^ were attributable to asymmetric and symmetric stretching vibrations of CH_2_ groups, while the signals at 2950 and 2850 cm^−1^ were due to the asymmetric and symmetric stretching vibrations of CH_3_. The peak at 1456 cm^−1^ indicates the asymmetric CH_3_ vibrations or CH_2_ scissor vibrations, while the signal at 1375 cm^−1^ was the result of the symmetric CH_3_ deformation [22]. All mentioned signals represent typical signals for polypropylene materials.

When comparing the FTIR spectrum of the ear loops with the spectrum of the synthetically produced polyurethane, it was found that the signals overlap at the following wavenumbers: 3290, 1700, 1630, and 1540 cm^−1^ [40]. There are minor differences, most likely due to the use of different additives.

The FTIR spectrum of the nose pads gave only two signals in the range from 3000 to 2800 cm^−1^, namely, at the wavenumber 2914 and 2847 cm^−1^, signals at 1471 and 1462 cm^−1^, small peak at 874 cm^−1^, and two signals at 729 and 718 cm^−1^. These signals are attributed to polyethylene [22].

**Figure 8:**
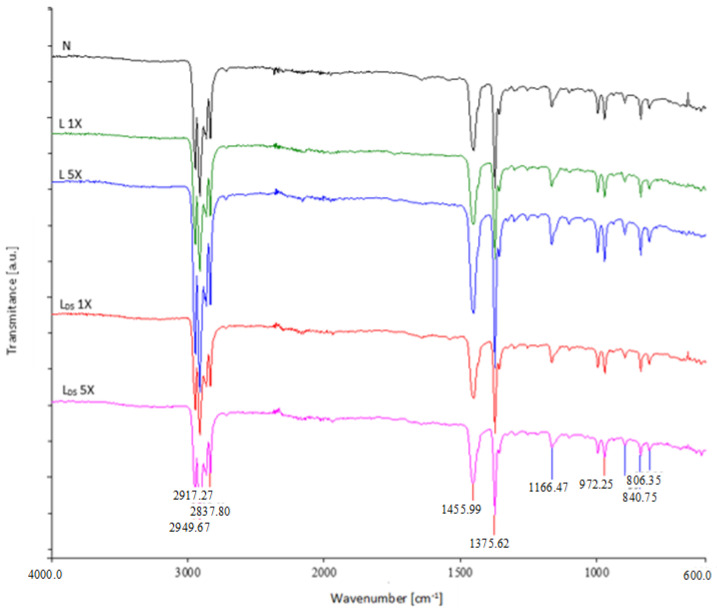
FTIR spectra of the front nonwoven layers of the medical mask before and after the first and fifth washing cycle.

**Figure 9:**
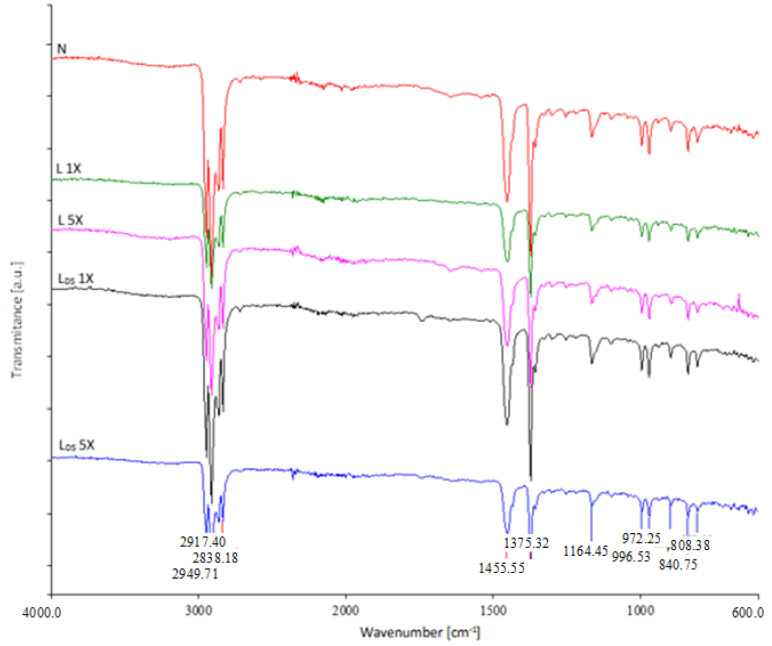
FTIR spectra of the back nonwoven layers of the medical mask before and after the first and fifth washing cycle.

Figure 8 shows the spectra of the front nonwoven layers of the medical mask before and after the first and fifth washing cycle with detergent in two washing variants, whereas Figure 9 shows the spectra of the back nonwoven layers of the medical mask before and after the first and fifth washing cycle with detergent in two washing variants.

According to the FTIR analysis performed, it was found that the washed (back and face) nonwoven layers of the medical mask do not indicate the influence of detergent or disinfectant on the chemical structure of the nonwoven layers in washing.

### 3.2. Characterization of the Aqueous Extract

In addition, the pH of the aqueous extract was tested. Variations in the preparation of the sample were made, leaving a portion of the medical masks whole and the other cut into strips (U), Table 8 and Table 9.

The aqueous extract of the whole mask samples before and after washing depends on the washing conditions. The aqueous extract of medical masks washed at home through one to five cycles has a different pH. A sample of the medical mask washed through five cycles has an almost 1.8 pH units higher value than after a single wash. The aqueous extract of medical masks washed through one to five cycles under laboratory conditions is alkaline, with all the samples having almost the same value (pH 8.9).

Washing with a standard detergent increased the pH of the water extract, which was slightly higher than the limits of the control system for laundries RAL-GZ 992 (RAL Gütezeichen RAL-GZ 992 Sicherheit durch professionellen Wäscheservice) [41].

Table 9 shows the pH results of the aqueous extract for the medical mask samples cut into strips (U).

The pH of the aqueous extract of the cut mask samples before washing at the same temperature was 0.5 pH units lower than that of the whole mask sample. The obtained results show that the washed cut mask samples have a more alkaline aqueous extract compared to the whole mask samples, which could be due to residual alkalis within the nonwoven structure, which has a higher migration potential away from the structure when the samples are cut.

#### 3.2.1. Surfactants Residuals Characterization

In accordance with the hypotheses highlighted in [19], decontamination methods should not only demonstrate effective pathogen reduction but also preserve the properties of medical masks without harmful chemical effects for the user. In accordance with that, the whole and cut mask samples were analyzed for the presence and content of surfactants before and after the first and fifth wash cycle, depending on the composition of the applied detergents and DDAC.

Medical masks not subjected to washing (N) were analyzed by determining all surfactants, ionic (anionic and cationic), and nonionic.

The home-washed samples were analyzed by determining the anionic and nonionic surfactants, while the laboratory-washed samples with the addition of DDAC in the rinse were analyzed by determining the anionic, cationic, and nonionic surfactants.

The results of potentiometric determination of residues of anionic surfactants in the aqueous extract of whole medical masks before and after washing are shown in Table 10.

The results of the amount of surfactants in the whole mask samples, Table 10, show the presence of ionic and nonionic surfactants only in some samples. The whole mask samples contained all surfactants before washing, with an extremely high amount of anionic surfactants and a much lower amount of nonionic and cationic surfactants compared to the anionic ones. The reason for this can indeed be attributed to contamination during production/packaging and/or an analysis error. The obtained amounts of cationic and nonionic surfactant per mass of the sample do not differ significantly, and in terms of residuals their values are lower than the permissible values according to the quality system, RAL-GZ 992.

In the 1× home-washed sample (H 1×), the anionic surfactant was isolated. Cumulative washing cycles had no effect on the increase in the residual amount of surfactants but eliminated them completely (H 5×).

There was a change after the laboratory washing of the whole mask samples compared to the unwashed whole mask samples, which can be concluded from the presence of the anionic surfactant in the 1× washed sample (L 1×) and a nonionic surfactant in the 5× washed sample (L 5×). Washing in the laboratory with a standard detergent and rinsing with the addition of DDAC (L_DS_) affected the state of the surface so that anionic and cationic surfactants were present after the first and fifth cycles, and their amount increased with the number of cycles, indicating the cumulative aggregation of surfactants.

Considering the potential and actual interactions of ionic surfactants (anionic and cationic) in the same bath, their combination on textiles is not common. However, in the performed procedure, where DDAC was added as a cationic surfactant in the fourth rinse cycle (after washing with standard detergent and the third rinse cycle in water), it partially reduced the amount of anionic surfactant, without eliminating it completely.

A conceptually identical analysis was performed on the cut mask samples (U), with the combined results of the surfactant analysis on the medical mask samples presented in Table 11.

Surfactant residuals were found in the unwashed and washed samples, and their values were within the limits set by the control system RAL-GZ 992. It is better to perform the analysis of the parameters on whole samples than on cut samples. The surfactant content results of the cut medical mask samples listed in Table 11 are significantly different from those listed in Table 10. Cationic surfactants were found only in the unwashed medical mask sample, and their amount was twice smaller than for the whole sample. According to this analysis, the cationic surfactant identified in the unwashed sample of the whole and cut medical masks indicates the possible addition of this surfactant during production as an additive to provide antimicrobial activity. The anionic surfactant was found on all the cut samples of the medical masks, and its amount was below the permissible levels (400 µg/g) according to RAL-GZ 992.

The nonionic surfactant was found in three samples (L U 1×, L_D_S U 1×, L_D_S U 5×), and its amount was not higher than the permissible amount (200 µg/g) according to RAL-GZ.

In summary, the FTIR analysis results confirmed that the back and front nonwoven layers of medical masks were made of polypropylene, and that the washing procedures had an unfavorable influence on the spectral values of the medical masks (fastness evaluation 3–4), through which the aesthetic properties of the masks were slightly damaged.

The new packaged masks are microbiologically safe; significant contamination occurs during home washing, whereas it is considerably lower during laboratory washing. The addition of a disinfectant in the fourth rinse has a favorable effect on microbiological efficiency, and no microorganisms are present. The increase in the pH of the surface of the medical masks washed through five cycles under laboratory conditions is due to the alkalinity of the standard detergent solution. The aqueous extract of the medical masks washed at home and under laboratory conditions through five cycles is alkaline, with almost the same value (pH 8.9) found for all samples.

It is better to perform the analysis on the whole samples than on the cut ones.

The nonwoven structure of the medical mask and the wrinkles that form during washing make it difficult to perform the analysis.

**Figure 10:**
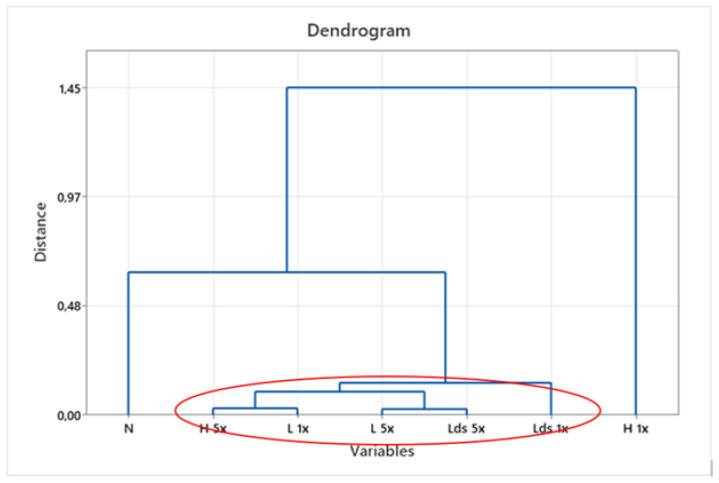
Dendrogram of Euclidean distance for contact angle, air permeability, and whiteness as variables of unwashed and 1× and 5× washed samples. Red circle indicates samples with very small distances.

By applying the hierarchical cluster analysis (HCA) [42] to the results of air permeability, contact angle, and whiteness of the samples, a group distribution was obtained, Figure 10.

The group distribution highlights the unwashed sample, and there is a greater distance between the samples washed 1× in the household washing machine. The highlighting of these samples confirms the earlier claim about the significantly different influence of the mechanics of the household washing machine compared to the laboratory washing device, Figure 10. The influence of the number of washing cycles on the observed parameters was additionally confirmed.

The similarities and differences between the groups show and confirm the claims about the impact of washing on the contact angle, which primarily depends on the number of wash cycles performed.

The static contact angle, air permeability, and FTIR results demonstrated the reusability and washability of medical masks at 50 °C in home and laboratory washing procedures. It was also confirmed that washed masks contain minimal amounts of residue, so contact with skin is not hazardous. The spectral properties of the washed masks changed slightly due to bleeding of the color during the washing process. The added benefit of the proposed approach was the use of the disinfectant didecyldimethylammonium chloride in the washing process.

## 4. Conclusions

This work is focused on the evaluation of different procedures of washing medical masks with detergents and the disinfectant DDAC. One of the hypotheses of this work was that medical masks can maintain filtration properties and breathability after washing at 50 °C.

The hydrophobicity of medical masks determined through the static contact angle depends on the number of cycles carried out. The static contact angle of the samples after the first cycle is lower than after the fifth cycle in all procedures. Images of ultrapure water drops on the surface confirm the hydrophobicity of the front/back of the medical mask before and after washing. The air permeability of washed samples in household and laboratory washing procedures through five cycles increases (~100 L/m^2^s) compared to the unwashed sample (~30 L/m^2^s). The spectral values of the washed medical masks confirmed slightly impaired aesthetic features.

The obtained results indicate that the performed washing procedures adversely affect some properties of the analyzed medical masks. Despite this, the washability of medical masks at 50 °C gives a basis for their reuse and the partial reduction in mask waste, whose disposal has not been adequately resolved.

The application of the disinfectant didecyldimethylammonium chloride in rinsing proved to be a useful method for maintaining the initial protective properties of medical masks.

## Figures and Tables

**Table 1:** Labeling of medical masks.

Label	Medical mask
N	Pristine
U	Cut
L 1×	Laboratory-washed—1 cycle
L 5×	Laboratory-washed—5 cycles
L_DS_ 1×	Laboratory-washed with addition of DDAC—1 cycle
L_DS_ 5×	Laboratory-washed with addition of DDAC—5 cycles
H 1×	Home-washed—1 cycle
H 5×	Home-washed—5 cycles

**Table 2:** Average values of spectral properties of the front surface of medical masks after the first and fifth washing cycle compared to the unwashed sample.

Sample	dL*	dC*	dH*	*dE*	ISO-A05	AATCC
H 1×	−0.447	1.383	0.269	1.573	4	4
H 5×	1.567	−4.083	−0.141	4.526	3	3
L 1×	−2.087	1.888	−0.219	3.291	3	3
L 5×	−0.907	−0.015	−0.782	2.076	4	4
L_DS_ 1×	0.028	−2.190	−0.722	2.341	4	3–4
L_DS_ 5×	−0.515	−0.264	−0.777	1.655	4	4

**Table 3:** Average whiteness of the back of medical masks before and after washing.

Sample	W_CIE_	TV	TD	Y
N	98.5	5.7	-	80.1
H 1×	98.8	5.4	-	81.0
H 5×	99.8	7.6	GG	77.1
L 1×	86.8	7.7	GG	72.3
L 5×	86.5	7.3	GG	73.4
L_DS_ 1×	86.1	6.5	GG	74.2
L_DS_ 5×	87.1	7.7	GG	73.0

**Table 4:** The average SCA of the liquid droplet with the front and back surface of the medical mask before and after washing.

SCA (°)							
Sample	N	H 1×	H 5×	L 1×	L 5×	L_DS_ 1×	L_DS_ 5×
Front surface	131.24	117.38	122.11	118.04	123.61	115.64	129.28
Back surface	122.30	109.48	113.78	123.02	117.77	123.84	112.46

**Table 5:** Air permeability of medical masks before and after washing.

Sample	N	H 1×	H 5×	L 1×	L 5×	L_DS_ 1×	L_DS_ 5×
v (L/m^2^s)	31.0	181.0	90.0	87.3	104.8	109.5	98.4

**Table 6:** pH value of the front surface of whole medical masks before and after washing.

Sample	pH	T (°C)
N	5.79	27.68
H 1×	5.72	27.68
H 5×	5.69	27.7
L 1×	5.07	25.3
L 5×	6.97	22.8
L_DS_ 1×	5.05	25.5
L_DS_ 5×	6.97	23.2

**Table 7:** pH value of the back surface of medical masks before and after washing.

Sample	pH	T (°C)
N	5.75	27.68
H 1×	5.71	27.73
H 5×	5.71	28.85
L 1×	5.06	25.6
L 5×	7.00	23.23
L_DS_ 1×	5.05	25.8
L_DS_ 5×	6.87	22.7

**Table 8:** pH of the aqueous extract of the whole mask samples before and after washing.

Sample	pH	T (°C)
N	7.01	23.6
H 1×	6.90	23.9
H 5×	8.74	25.2
L 1×	8.87	23.7
L 5×	8.95	23.1
L_DS_ 1×	8.93	23.9
L_DS_ 5×	8.81	22.9
KCl	5.73	23.8

**Table 9:** pH of the aqueous extract of medical mask samples cut into strips before and after washing.

Sample	pH	T (°C)
N U	6.49	23.9
H U 1×	8.01	24.4
H U 5×	8.63	24.8
L U 1×	9.12	23.9
L U 5×	8.72	22.9
L_DS_ U 1×	8.97	23.9
L_DS_ U 5×	9.21	22.7
KCl	5.91	22.7

**Table 10:** Amounts of anionic, nonionic, and cationic surfactants in the aqueous extract of medical masks before and after washing—whole samples.

Sample	Surfactant Residuals in the Sample (μg/g)		
	Anionic	Nonionic	Cationic
N	1175.98	68.42	46.32
H 1×	361.01	-	-
H 5×	-	-	-
L 1×	-	348.9	-
L 5×	50.10	-	-
L_DS_ 1×	112.5	-	44.24
L_DS_ 5×	284.43	-	130.14

**Table 11:** Amounts of anionic, nonionic, and cationic surfactants in the aqueous extract of cut medical masks before and after washing.

Sample	Surfactant Residuals in the Sample (µg/g)		
	Anionic	Nonionic	Cationic
N U	-	-	28.24
H U 1×	201.42	-	-
H U 5×	339.47	-	-
L U 1×	34.00	110.75	-
L U 5×	281.95	-	-
L_DS_ U 1×	91.73	93.31	-
L_DS_ U 5×	285.17	196.83	-

## Data Availability

Not applicable.
